# Dynamic gel as artificial interphase layer for ultrahigh-rate and large-capacity lithium metal anode

**DOI:** 10.1038/s41467-023-39636-6

**Published:** 2023-07-07

**Authors:** Chao Chen, Jiaming Zhang, Benrui Hu, Qianwen Liang, Xunhui Xiong

**Affiliations:** School of Environment and Energy, Guangdong Provincial Key Laboratory of Advanced Energy Storage Materials, South China University of Technology, Guangzhou, 510640 P. R. China

**Keywords:** Batteries, Batteries, Batteries

## Abstract

Constructing a stable artificial solid-electrolyte interphase has become one of the most effective strategies to overcome the poor reversibility of lithium metal anode, yet the protection role is still insufficient at elevated current densities over 10 mA cm^−2^ and large areal capacities over 10 mAh cm^−2^. Herein, we propose a dynamic gel with reversible imine groups, which is prepared *via* a cross linking reaction between flexible dibenzaldehyde-terminated telechelic poly(ethylene glycol) and rigid chitosan, to fabricate a protective layer for Li metal anode. The as-prepared artificial film shows combined merits of high Young’s modulus, strong ductility and high ionic conductivity. When the artificial film is fabricated on a lithium metal anode, the thin protective layer shows a dense and uniform surface owing to the interactions between the abundant polar groups and lithium metal. Besides, the polar groups in the artificial film can homogenize the distribution of Li^+^ at the electrode/electrolyte interface. As a result, cycle stability over 3200 h under an areal capacity of 10 mAh cm^−2^ and a current density of 10 mA cm^−2^ has been obtained for the protected lithium metal anodes. Moreover, cycling stability and rate capability has been also improved in the full cells.

## Abstract (web summary)

Achieving stable lithium metal anodes under large current densities over 10 mA cm^−2^ and area capacities over 10 mAh cm^−2^ remains a critical challenge. Here, authors propose a dynamic gel as protect layer for lithium metal anode to facilitate with the Young’s modulus, flexibility and ionic conductivity, thus to stabilize the lithium metal anode.

## Introduction

The rapid development of electric vehicles in the recent years imposes increasing researches on high-energy-density lithium-ion batteries (LIBs). Among different anode candidates, Li metal has attracted numerous attentions due to its high theoretical specific capacity (3860 mAh g^−1^) and the lowest redox potential (−3.04 V)^1–3^. However, the poor reversibility and Li dendrite growth hinder the commercial applications of Li metal anode. Because of the active chemical properties, Li metal can react with solvents and Li salts in the organic electrolytes to form a thin solid electrolyte interface (SEI) layer on surface to prevent the continuous side reactions^4–7^. However, the fragile SEI film cannot accommodate the huge volume changes during the charge/discharge processes, and the cracks in the SEI will aggravate the parasitic reactions between the fresh Li metal and electrolyte, resulting in the rapid deterioration of Coulombic efficiency (CE) and cycle life of the Li metal batteries (LMBs). Besides, the non-uniformity of the native SEI film in structure and composition will induce the uneven deposition of Li ions, which will lead to the generation of Li dendrites and even safety hazards^8–11^. These concerns can be accelerated when the LMBs are operated at elevated current densities and areal capacities. Therefore, constructing an artificial SEI layer to replace the native SEI has been proposed to stabilize the Li metal anode.

An ideal artificial SEI layer has been emphasized to possess excellent chemical stability to prevent the reaction between Li metal and electrolyte, high ionic conductivity to homogenize the Li ions transfer^2,12–14^. Besides, the mechanical properties of the artificial SEI layer, especially the Young’s modulus and flexibility, also play a critical role in stabilizing the Li metal anode^7,15–18^. A high Young’s modulus is desirable to block the continually growth of Li dendrites, while a superior flexibility is indispensable to accommodate the severe volumetric variations and avoid the generation of cracks in the SEI layer during the repetitive cycles. However, it remains a critical challenge to simultaneously achieve high Young’s modulus and strong flexibility for artificial SEI layers. An inorganic SEI layers has ultrahigh Young’s modulus, while the brittleness nature and poor contact with Li metal cannot guarantee a long-term stable cycling at large areal capacities^19,20^. The polymer SEI layers can effectively buffer the volume changes than inorganic SEI layers during the stripping/plating processes. However, they suffer from insufficient mechanical modulus and cannot suppress the growth of Li dendrite. Besides, the poor ionic conductivity of polymer SEI layers results in nonuniform Li^+^ diffusion and uneven Li deposition^21,22^. To overcome these problems, incorporating the inorganic components into polymer matrixes to fabricate hybrid SEI layers has been proposed^23–25^. The hybrid SEI layers inherit the individual advantages of two components and demonstrate suitable mechanical strength and flexibility to stabilize the Li metal anode. However, the nanostructured inorganic components in the hybrid SEI layers show notable tendencies to agglomerate because of the high surface energy as well as the inferior compatibility with polymers^26^, which will induce the uneven distribution of Li^+^ flux and the inhomogeneous Li deposition. Accordingly, most of the Li anodes protected by the hybrid SEI layers cannot deliver satisfactory stability at high current density (≥10 mA cm^−2^) and large areal capacity (≥10 mAh cm^−2^). Therefore, it remains a critical challenge to develop advanced SEI layers that simultaneously possess adequate mechanical properties as well as high ionic conductivity to achieve stable Li metal anode under high current density and areal capacity.

Herein, we propose a dynamic gel to fabricate a robust artificial layer to achieve stable Li metal anode under both ultrahigh current densities and areal capacity. The dynamic gel is prepared through a cross-linking reaction between chitosan (CS) and dibenzaldehyde-terminated telechelic poly(ethylene glycol) (DF-PEG-DF) in dimethyl sulfoxide (DMSO) solvent. Benefiting from the flexible polyethylene glycol (PEG) backbone and the ionic-conductive, rigid chitosan component, the CS/DF-PEG-DF SEI layer is endowed with adequate ductility, favorable mechanical strength and high ionic conductivity. Furthermore, the rich polar groups in the CS provide a large number of interaction sites for the efficient adhesion to Li metal and homogeneous distribution of Li^+^ at the Li/electrolyte interface. As a result, the CS/DF-PEG-DF coated Li anode (CS/DF-PEG-DF@Li) shows an unprecedented plating/stripping cyclability more than 3200 h at both high current density of 10 mA cm^−2^ and large areal capacity of 10 mAh cm^−2^ in symmetrical cells. Besides, even under ultrahigh current density of 50 mA cm^−2^ and areal capacity of 50 mAh cm^−2^, the CS/DF-PEG-DF@Li anode delivers a long-term stability over 600 h. Furthermore, when coupled with high-loading LiFePO_4_ (LFP) cathode (~20 mg cm^−2^), the CS/DF-PEG-DF@Li anode demonstrates dramatically enhanced cycle performance and rate capability than the bare Li counterpart.

## Results

### Preparation and characterization of the dynamic gel

**Fig. 1:**
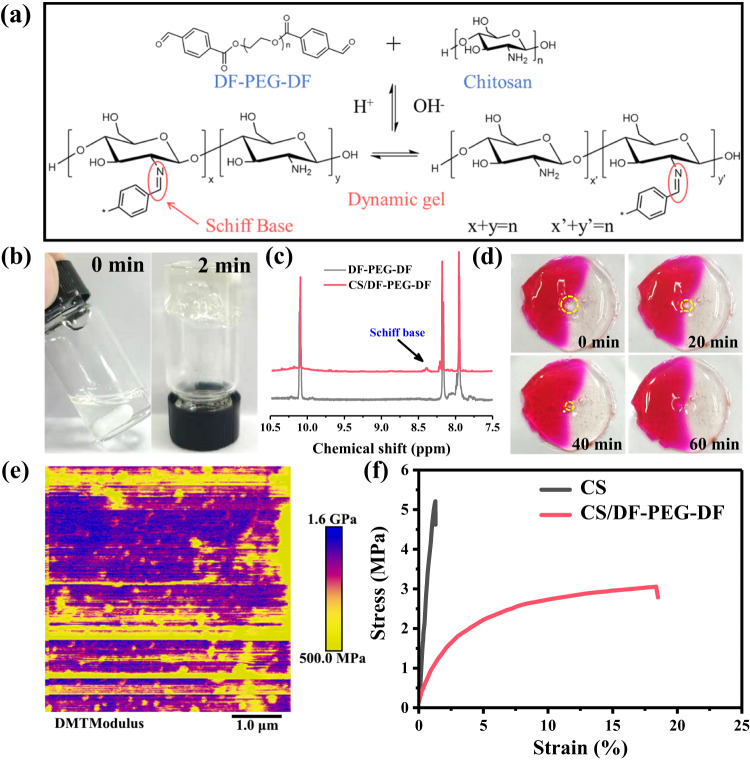
The design and mechanical property of CS/DF-PEG-DF film. **a** Schematic diagram of the cross-linking reaction between DF-PEG-DF and CS to form gel. **b** Photographs of the CS/DF-PEG-DF gel formation process. **c** ^1^H NMR spectrum of DF-PEG-DF and DF-PEG-DF with CS in CDCl_3_. **d** The images of self-healing process of CS/DF-PEG-DF at room temperature. **e** Young’s modulus mapping of CS/DF-PEG-DF film. **f** Stress-strain curves of CS and CS/DF-PEG-DF film.

As shown in Fig. 1a, the CS/DF-PEG-DF dynamic gel was prepared by cross linking the chitosan and DF-PEG-DF *via* a rapid Schiff reaction in DMSO solvent. The solvent forms a gel solid after mixing for two minutes (Fig. 1b), which is caused by the reaction between the amino group in chitosan and the benzaldehyde group in DF-PEG-DF. To verify the speculation, ^1^H NMR spectra of DF-PEG-DF and CS/DF-PEG-DF were studied. Compared with DF-PEG-DF, CS/DF-PEG-DF shows an obvious peak of Schiff base at the chemical shift of 8.37 ppm (Fig. 1c and Supplementary Fig. 1)^27,28^. The existence of reversible imine bonds in Schiff base endows the CS/DF-PEG-DF with excellent self-healing ability, which can be observed from the gradual closing hole in the center of the gel (Fig. 1d). The superior self-healing ability of CS/DF-PEG-DF gels is also demonstrated by testing the rheological properties of the CS/DF-PEG-DF gels (Supplementary Fig. 2). When the CS/DF-PEG-DF gel was dried at 30 °C for 12 h in a glove box, CS/DF-PEG-DF film with DMSO content of 5.1 wt.% was obtained (Supplementary Fig. 3), and then the physicochemical properties of as-obtained film were evaluated. Arising from the high Young’s modulus of CS (2.3 GPa, Supplementary Fig. 4a), CS/DF-PEG-DF film shows a high mechanical strength of 1.0 GPa (Fig. 1e), much higher than that of DF-PEG-DF (164 MPa, Supplementary Fig. 4b). Tensile tests were also performed to study the ductility of CS/DF-PEG-DF film (Fig. 1f). Compared with the brittle nature of CS, the elongation at break of CS/DF-PEG-DF can reach up to 18.5%. Obviously, CS/DF-PEG-DF film displays a combined merit of superior ductility and high mechanical strength, which is desired for the protective layer for Li metal anode.

Meanwhile, the electrochemical impedance spectroscopy (EIS) measurements show that the CS/DF-PEG-DF film exhibits a high ion conductivity of 9.33 × 10^−5^ S cm^−1^ at room temperature (Supplementary Fig. 5), which is much higher than that of PEO (10^−10^ S cm^−1^)^29^, as well as 4−5 orders of magnitude higher than the natural SEI components LiF (10^−9^ S cm^−1^) and Li_2_CO_3_ (10^−8^ S cm^−1^)^30,31^. The ultra-high ionic conductivity of the CS/DF-PEG-DF film is attributed to the hydroxyl group on CS, which can facilitate Li^+^ transfer by forming a continuous hopping pathway for Li^+^ and prevent the migration of PF_6_^-^ anions *via* hydrogen bonds^32^. Besides, the PEG grafting can break the hydrogen bonds in the CS molecule, releasing more amino and hydroxyl groups on the chitosan backbone to interact with the anion and then accelerate the Li^+^ transfer^33,34^. This phenomenon is also confirmed by the effect of PEG grafting on the Li-ion transference number (*t*_*Li+*_) of CS/DF-PEG-DF. As shown in Supplementary Fig. 6, the *t*_*Li+*_ of CS/DF-PEG-DF is calculated to be 0.76, much higher than that of PEO (0.48)^35^. When CS/DF-PEG-DF is proposed as an artificial layer for Li metal anode, the superior ionic conductivity with high Li-ion transference number can effectively reduce the interface impedance and alleviate the uneven ion flux.

### Characterization of the artificial SEI layer

**Fig. 2:**
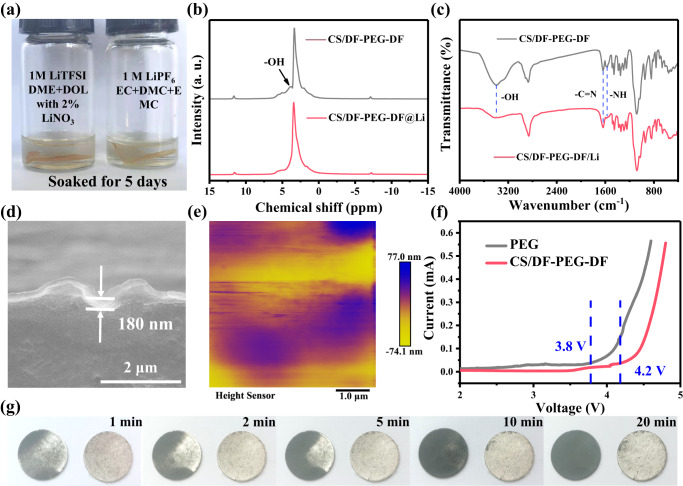
Reaction mechanism and chemical stability of CS/DF-PEG-DF@Li. **a** The chemical stability of CS/DF-PEG-DF film in different electrolytes. **b** ^1^H SSNMR spectra and (**c**) FTIR spectra of CS/DF-PEG-DF and CS/DF-PEG-DF/Li. **d** Cross-section SEM image and (**e**) AFM image of CS/DF-PEG-DF@Li formed by adding 50 μL CS/DF-PEG-DF/DMSO solution. **f** Linear sweep voltammetry (LSV) curves of Li||PEG or CS/DF-PEG-DF||stainless steel (SS) batteries. **g** Photographs of the bare Li and CS/DF-PEG-DF@Li anode exposed in ambient air for different minutes.

To further clarify whether the CS/DF-PEG-DF film is suitable for artificial SEI layer for Li metal anode, the chemical stability of CS/DF-PEG-DF film was also investigated. As shown in Fig. 2a and Supplementary Fig. 7 and 8, DF-PEG-DF is soluble in both ester and ether electrolyte, however, CS/DF-PEG-DF film demonstrates excellent chemical stability in both of two electrolytes. Therefore, the cross-linking reaction can also remarkably change the chemical properties of individual components. Then the chemical stability of CS/DF-PEG-DF film with Li metal was evaluated. After sandwiched by two Li foils, the chemical-structure change of the as-obtained film (CS/DF-PEG-DF/Li) was studied with the solid-state ^1^H nuclear magnetic resonance (^1^H SSNMR) and Fourier transform infrared spectroscopy (FT-IR). As shown in Fig. 2b, the peak at 4.0 ppm in CS/DF-PEG-DF film can be assigned to the -OH in CS^36,37^, and the absence of this peak in CS/DF-PEG-DF-Li film suggests that the hydroxyl group in CS is consumed by the Li metal. The FT-IR spectroscopy in CS/DF-PEG-DF film shows strong intensity of the -NH_2_ and -OH groups, which are located at 1569 and 3365 cm^−1^ (Fig. 2c), respectively^38–40^, while a great decrease of these two groups can be observed in the CS/DF-PEG-DF/Li film. Meanwhile, the characteristic peak at 1636 cm^−1^ in CS/DF-PEG-DF/Li film, which can be indexed to the -C = *N* group^41^, is well maintained and experiences no obvious changes. Therefore, the ^1^H SSNMR and FT-IR results demonstrate that the chemical reaction with Li metal is mainly occurred at -OH and -NH_2_ groups in CS/DF-PEG-DF film.

The high Young’s modulus, superior ductility, ultrahigh ionic conductivity and excellent chemical stability in the electrolyte enable CS/DF-PEG-DF film to be a promising protective layer for Li metal anode. To fabricate a thin CS/DF-PEG-DF film on surface of Li metal, CS solution and DF-PEG-DF solution in DMSO were mixed with a volume ratio of 1:1 by magnetic stirring for tens of seconds and then drop-coated on a Li foil immediately. The solution on Li metal becomes a transparent gel within two minutes, and CS/DF-PEG-DF@Li anode is obtained after DMSO evaporation at 30 °C for 12 h. Benefiting from the abundant polar groups in CS/DF-PEG-DF, the protective layers in various thicknesses from 180 to 1230 nm are strongly adhered to the Li metal and show smooth and dense surface morphology (Fig. 2d and Supplementary Fig. 9 and 10), which can be confirmed by the atomic force microscope (AFM) analysis. As exhibited in Fig. 2e, the thin CS/DF-PEG-DF protection layer on Li metal shows the root mean square roughness (Rq) and average roughness (Ra) are as low as 20.2 and 16.1 nm, respectively. Besides, the dense feature of the protective layer can be verified by enhanced air stability of CS/DF-PEG-DF@Li anode. When bare Li and CS/DF-PEG-DF@Li anode are exposed in air with a humidity of about 40%, the bare Li turns to black after 1 min (Fig. 2g). However, the CS/DF-PEG-DF@Li anode can keep the metallic luster for 20 min under the same condition. Furthermore, the CS/DF-PEG-DF protective layer with DMSO content of 0.6 wt.% retains a high mechanical strength of 1.1 GPa after reaction with Li metal (Supplementary Figure 3b and Fig. 11a). After immersion in the electrolyte for 24 h, the mechanical strength of protective layer experiences a slight drop to 810 MPa (Supplementary Fig. 11b). Nevertheless, the ductility of CS/DF-PEG-DF film is well maintained after reaction with Li and even undergoes an obvious increase after immersed in electrolyte, as shown in Supplementary Fig. 12. Linear sweep voltammetry (LSV) curve indicates that the electrochemical stability of CS/DF-PEG-DF artificial layer is increased to 4.2 V (Fig. 2f), much higher than that of PEG layer (3.8 V), suggesting that CS/DF-PEG-DF@Li anode can be paired with high-voltage cathode to achieve high energy density.

### Li plating/stripping performance of symmetric cells

**Fig. 3:**
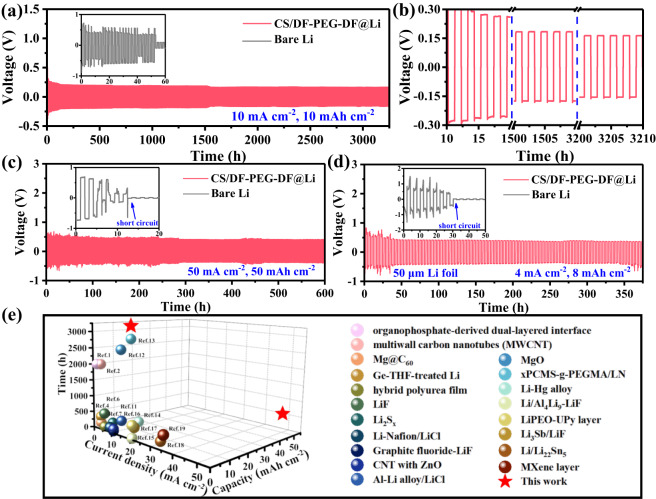
Electrochemical performance of symmetric Li||Li cells. **a** Voltage profiles of bare Li and CS/DF-PEG-DF@Li anode in symmetric cell at 10 mA cm^−2^ under a fixed capacity of 10 mAh cm^−2^. **b** The corresponding enlarged voltage profiles of CS/DF-PEG-DF@Li anode for different cycles. **c** Voltage profiles of bare Li and CS/DF-PEG-DF@Li anode in symmetric cell at current density of 50 mA cm^−2^ and high capacity of 50 mAh cm^−2^. **d** Cycle stability of symmetrical cells employing bare Li and CS/DF-PEG-DF@Li with thickness of 50 μm at 4 mA cm^−2^ under a fixed capacity of 8 mAh cm^−2^. **e** Comparison of maximum current density and the corresponding cycle life of the CS/DF-PEG-DF@Li with the representative protected Li metal anode (Data is obtained from Table S1 in Supporting Information).

To demonstrate the capability of CS/DF-PEG-DF protective layer in enhancing the interfacial stability of Li metal anode, the stripping/plating cycling in the symmetric cells in ether electrolyte were studied. The symmetric cells employing CS/DF-PEG-DF@Li anode with different thicknesses of protective layers were fabricated to achieve the best electrochemical performances. As shown in Supplementary Fig. 13, DMSO has been demonstrated to react with Li metal, however, the reaction products on Li metal cannot improve the electrochemical performances of Li metal anode (Supplementary Fig. 14). Meanwhile, CS/DF-PEG-DF@Li anode with too thin or too thick artificial layers cannot deliver desirable cycling performances (Supplementary Fig. 15). When the thickness of protective layer was set at 180 nm, CS/DF-PEG-DF@Li anode demonstrates the longest cycle life. Specifically, the symmetric cell with CS/DF-PEG-DF@Li anode exhibits an unprecedented cycle stability over 3200 h under a high areal capacity of 10 mAh cm^−2^ at 10 mA cm^−2^ (Fig. 3a). The regular yet flat voltage profiles as well as the stable overpotential in the whole cycling processes illustrate the rapid Li^+^ transfer kinetics and favorable interfacial stabilities (Fig. 3b). In vast contrast, the cells with bare Li anode show higher overpotentials over 500 mV with severe voltage oscillation, and internal short-circuit is observed after cycling for 54 h owing to the growth of Li dendrites. The substantially enhanced interfacial stability of CS/DF-PEG-DF@Li anode is also proved by the electrochemical impedance spectroscopy during the cycling (Supplementary Fig. 16), which shows the interfacial impedance of CS/DF-PEG-DF@Li electrode remains stable after 10 cycles.

Except for the exceptional cycling stability, the CS/DF-PEG-DF@Li anode also delivers extraordinary rate capabilities. When the current density was dramatically increased to 50 mA cm^−2^, the symmetrical cell with CS/DF-PEG-DF@Li anode can maintain a low and steady overpotential at 276.2 mV for 800 h (Supplementary Fig. 17). Furthermore, the cell with CS/DF-PEG-DF@Li anode can even run for 600 h with a steady overpotential of 400.4 mV at a large areal capacity of 50 mAh cm^−2^ at 50 mA cm^−2^ (Fig. 3c). In striking contrast, huge fluctuations are emerged in voltage curves for the cell with bare Li anode in the initial cycles and the cell failure happens within 11 h. To verify the protection role of CS/DF-PEG-DF layer, the electrochemical performances of a thin Li foil (~50 μm) protected by CS/DF-PEG-DF layer were also investigated. Impressively, stable Li plating/stripping for 380 h is achieved at a fixed capacity of 8 mAh cm^−2^ and a current density of 4 mA cm^−2^ (Fig. 3d). Considering the thin Li anode shows a capacity of 9.09 mAh cm^−2^ (Supplementary Fig. 18), the utilization ratio of metallic Li can reach up to 88.0%. To the best of our knowledge, the cycle stability and utilization rate of CS/DF-PEG-DF@Li anode stands at the highest level compared with the reported Li anodes protected by different artificial layers (Fig. 3e, Supplementary Table 2).

To clarify the role of the unique physicochemical properties of CS/DF-PEG-DF protection layer, the electrochemical performances of symmetric cells with DF-PEG-DF@Li or CS@Li anode were also studied. As shown in Supplementary Fig. 19, DF-PEG-DF@Li and CS@Li anodes can only maintain stable plating/stripping cycling for 150 h and 400 h under a capacity of 10 mAh cm^−2^ at 10 mA cm^−2^, respectively, which are much poorer than that of CS/DF-PEG-DF@Li anode. Besides, top-view SEM images in Supplementary Fig. 20 show that numerous cracks are observed on both DF-PEG-DF@Li and CS@Li anodes after 50 cycles. For DF-PEG-DF@Li anode, the DF-PEG-DF layer was firstly dissolved in the electrolyte, and then the complex reaction between Li^+^ and DF-PEG-DF can reform a protective layer *via* absorbing on the surface of Li metal^42^. The DF-PEG-DF-based artificial layer cannot effectively regulate the deposition of Li^+^ owing to the poor ionic conductivity, leading to the formation of Li dendrites at high current density. Meanwhile, the DF-PEG-DF-based layer has no adequate mechanical strength to withstand the high stress generated by the Li dendrites, and then a large number of dendrites appear at the cracks, (Supplementary Fig. 20a). For CS@Li anode, the high ionic conductivity of CS (10^−4^ S cm^−1^)^43^ can increase the uniformity of Li deposition, however, the rigid CS protective layer is still fractured (Supplementary Fig. 20b), which is attributed to the huge volume expansion at high areal capacity of 10 mAh cm^−2^. In sharp contrast, CS/DF-PEG-DF@Li still keeps a very smooth and dense surface without any protrusions or cracks (Supplementary Fig. 20c). It can be observed that the high ionic conductivity of protective layer can promote uniform deposition of Li^+^, however, the lack of flexibility will still cause fracture of artificial SEI due to volume expansion during cycling, especially at a large deposition capacity. Besides, the high mechanical strength is also highly desirable for protective layer to block the continuous growth of Li dendrites. Therefore, the combined merits of high mechanical strength, strong ductility and ultrahigh ionic conductivity of the CS/DF-PEG-DF artificial layer have made a critical role in improving the stability of Li metal anode.

### The role of artificial SEI layer in stabilizing Li metal anode

**Fig. 4:**
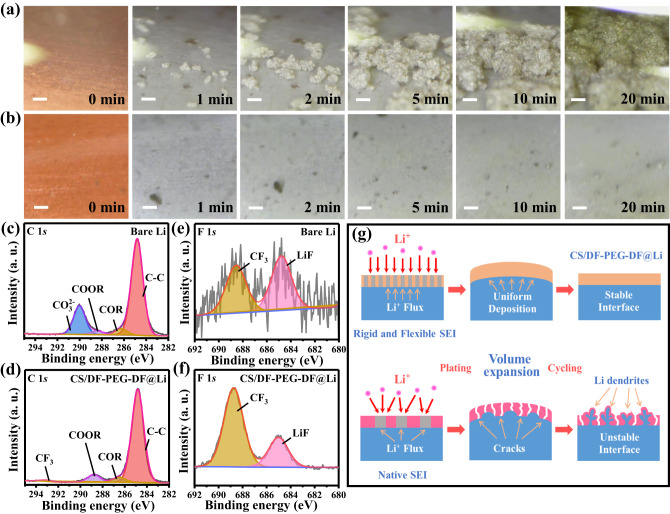
In situ Optical microscopy and SEI compositions of bare Li and CS/DF-PEG-DF@Li electrode. In-situ optical images of Li deposition on (**a**) bare Cu and (**b**) CS/DF-PEG-DF@Cu in Li||Cu cells at different minutes (Scale bar, 100 μm); C 1*s* XPS spectra of (**c**) bare Li and (**d**) CS/DF-PEG-DF@Li anodes after 10 cycles at 10 mA cm^−2^ under a capacity of 10 mAh cm^−2^; F 1*s* XPS spectra of (**e**) bare Li and (**f**) CS/DF-PEG-DF@Li anodes after 10 cycles at 10 mA cm^−2^ under a capacity of 10 mAh cm^−2^. **g** Schematic diagram of Li plating/stripping behaviors for the bare Li and CS/DF-PEG-DF@Li anode.

To gain more reason for the enhanced interfacial stability of CS/DF-PEG-DF@Li anode, in-situ optical microscopy was used to monitor the Li deposition process at a high current density of 10 mA cm^−2^ (Supplementary information Movie S1). As shown in Fig. 4a, Li dendrites are observed on the bare Cu foil after deposition for 1 min, and the Li dendrites keep gradual growth during the following 20 min. Notably, the Li deposition on the Cu foil coated by CS/DF-PEG-DF layer is homogenized. The CS/DF-PEG-DF@Cu electrode maintains a very smooth and uniform surface, and no dendrites are found in the whole deposition process (Fig. 4b). The regulated Li deposition can be attributed to the abundant hydroxyl groups in the CS/DF-PEG-DF. It has been reported that the interaction between the hydroxyl groups and Li^+^ can homogenize the distribution of Li^+^ at the molecular level and facilitate the layer-by-layer deposition of Li^+^^19,44^. Besides, the surface chemistries of cycled Li metal anodes were also studied *via* X-ray photoelectron spectroscopy (XPS) analysis to illustrate the advantage of CS/DF-PEG-DF layer. In the C 1*s* spectrum of bare Li after cycling in symmetric cells (Fig. 4c, d), the peak at 289.9 eV corresponds to CO_3_^2−^, which mainly comes from the decomposition product of solvent through the side reactions^45^. Obviously, the peak intensity of CO_3_^2−^ for bare Li is much stronger than that of CS/DF-PEG-DF@Li anode, indicating that the stable CS/DF-PEG-DF protective layer effectively can inhibit the penetration of the electrolyte and reduce the decomposition of the solvents. In the F 1*s* spectra in Fig. 4e, f, the peaks at 684.9 and 688.7 eV can be attributed to LiF from the decomposition of Bis(trifluoromethane)sulfonimide (LiTFSI) and -CF_3_ in LiTFSI^45^, respectively. It has been reported that the naturally generated SEI on Li metal contains two layers, including the inner inorganic layer and outer organic layer^46,47^. Owing to the continuous side reaction between the electrolyte and the bare Li metal, the cycled bare Li is covered by a thick layer of organic components, very weak signal of LiF can be observed in the F 1*s* XPS spectrum. Meanwhile, prior to XPS characterization, the cycled Li metal was rinsed with DOL solvent to remove the residual LiTFSI, resulting in a low amount of -CF_3_ on the cycled Li metal anode. However, a high content of LiF is observed on surface of CS/DF-PEG-DF@Li anode. According to the previous reports^48–50^, the remaining polar groups in CS/DF-PEG-DF layer can induce the break of C-F bond in LiTFSI *via* forming hydrogen bonds with LiTFSI, therefore forming a thin LiF layer on top surface CS/DF-PEG-DF@Li anode. The formed thin LiF layer during the initial cycles in the initial cycles can further enhance the chemical and structural stability of CS/DF-PEG-DF@Li anode in the following long-term cycles, which can be attributed to the ultra-high shear modulus, low surface diffusion barrier and high surface energy of LiF^47^. In the S 2*p* XPS spectra, the peak at 167.1 eV can be ascribed to Li_*x*_SO_*y*_ (Supplementary Fig. 21), a typical decomposition product of the LiTFSI, while the peak at 169.1 eV comes from the residual LiTFSI^45^. Similar with F 1*s* XPS spectra, the S 2*p* XPS spectra on the cycled bare Li metal anode are very weak. Therefore, XPS results show that the CS/DF-PEG-DF protective layer effectively reduces the consumption of electrolyte and enhances the stability of Li metal anode.

According to the above discussions, the unprecedented cycle stability at high current density of 10 mA cm^−2^ and large areal capacity of 10 mAh cm^−2^ can be summarized as following aspects (Fig. 4g). Firstly, the cross-linking reaction between CS and DF-PEG-DF achieves a balance between mechanical strength and ductility for the protective layer, which can block the continuous growth of Li dendrites and accommodate the volume changes during the stripping/plating processes. Secondly, the CS/DF-PEG-DF layer shows dense feature and strong interaction with Li metal owing to the rich polar groups, which can effectively prevent the side reaction at the Li/electrolyte interface. Besides, the abundant polar groups in CS/DF-PEG-DF layer can homogenize the distribution of Li^+^ at the interface *via* their interactions. Lastly, the ultrahigh ionic conductivity as well as high Li^+^ transference number can provide fast Li^+^ transfer path in the CS/DF-PEG-DF artificial layer. These synergetic effects can thereby uniform the Li deposition and enhance the interfacial stability of Li metal anode.

### Coulombic efficiency and full cells performance

**Fig. 5:**
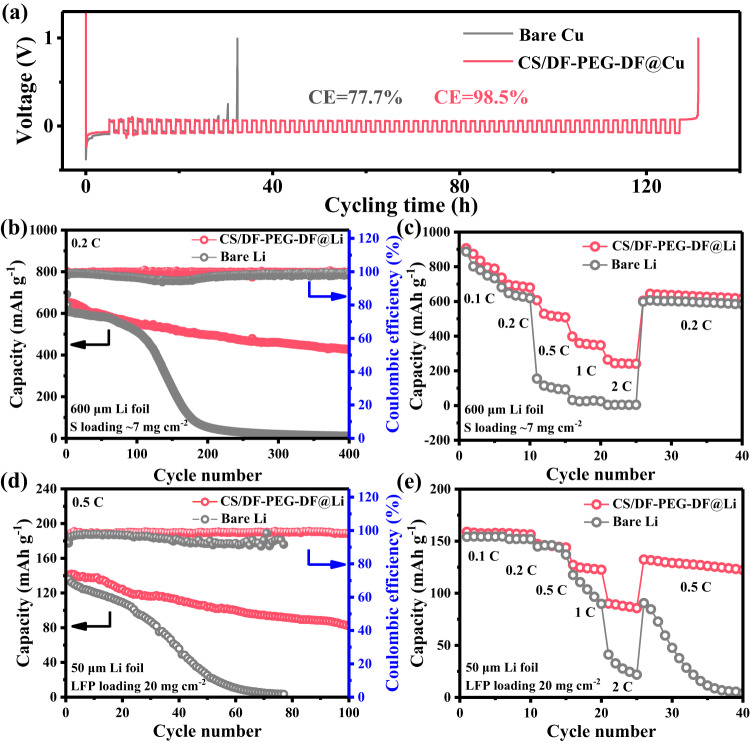
Electrochemical performance of Li||Cu half cells and Li||S/Li||LFP full cells. **a** CEs measured by Aurbach method of different electrodes in Li||Cu cells in ester electrolyte. **b** Cycle performances of Li||S and CS/DF-PEG-DF@Li||S cells at 0.2 C. **c** Rate performances at various C-rates of the Li||S and CS/DF-PEG-DF@Li||S cells. **d** Cycle performances of Li||LFP and CS/DF-PEG-DF@Li||LFP cells at 0.5 C. **e** Rate performances at various C-rates of the Li||LFP and CS/DF-PEG-DF@Li||LFP cells.

Encouraged by the significantly enhanced interfacial stability, the reversibility of CS/DF-PEG-DF@Li anode was also investigated by the CE test in Li||Cu cells, in which Cu foil coated by CS/DF-PEG-DF layer (~180 nm) was utilized as work electrode. The Aurbach method was used to simulate the behavior of Li plating/stripping under actual working conditions^51^, in which 5 mAh cm^−2^ Li was firstly deposited on Cu foil or CS/DF-PEG-DF@Cu, and then the cells were cycled at a current density of 1 mA cm^−2^ and a plating/stripping amount of 1 mAh cm^−2^ (Fig. 5a). For CS/DF-PEG-DF@Cu, the irreversible Li capacity of 1.01 mAh cm^−2^ is consumed after 60 cycles, and the average CE is 98.5%. On the other hand, the deposited Li on bare Cu was completely consumed at the 13th cycle, and the charge potential reaches to 1.0 V, with an average CE of 77.7%. The long-term reversibility of Li plating/stripping in LiǁCu batteries under extreme conditions was further analyzed. As shown in Supplementary Fig. 22a, the Li||CS/DF-PEG-DF@Cu battery maintains an average CE about 94.8% for 180 cycles at a current density of 1 mA cm^−2^ and a fixed capacity of 1 mAh cm^−2^. Besides, the Li||CS/DF-PEG-DF@Cu battery exhibits an almost constant voltage distribution with an overpotential of ~50 mV during 100 cycles (Supplementary Fig. 23). Even at higher current density and areal capacity, the Li||CS/DF-PEG-DF@Cu battery can keep a stable CE above 95.0% for 100 cycles (Supplementary Fig. 22b). In contrast, the CE of the Li||Cu battery with pristine Cu foil has dropped to 80% within 50 cycles, which shows that the Li metal without the protection of CS/DF-PEG-DF protective layer can continuously react with the electrolyte. The cycling CE results show that the CS/DF-PEG-DF protective layer on Li metal surface can suppress the side reactions between Li metal and electrolyte during the long-term cycling.

To investigate the potential application of the CS/DF-PEG-DF@Li anode in practical batteries, the protected Li metal anode was paired with different cathodes to assemble the full cells. The electrochemical performances of full cells constructed by self-made sulfur/carbon cathode (~7 mg cm^−2^) and CS/DF-PEG-DF@Li anode (600 μm) were firstly investigated. As shown in Fig. 5b and Supplementary Fig. 24a, the full cell with CS/DF-PEG-DF@Li anode delivers a capacity of 426.2 mAh g^−1^ after 400 cycles at 0.2 C, with a capacity retention rate of 61.6%. However, the full cell with bare Li shows a similar capacity at the initial cycles and shows a rapid decrease after 100 cycles. Under the protection of CS/DF-PEG-DF layer, the Li-S full cell also shows much higher capacities than that with bare Li anode at high current densities from 0.5 to 2 C (Fig. 5c). To demonstrate the protection role of artificial SEI layer at a more practical addition, the electrochemical performances of a thin CS/DF-PEG-DF@Li anode (50 μm) were also evaluated by coupling with a commercial high-loaded LiFePO_4_ electrode (20 mg cm^−2^). As shown in Fig. 5d and Supplementary Fig. 24b, Li||LFP cells assembled using bare Li and CS/DF-PEG-DF@Li electrodes in the ester electrolyte also display similar capacities in the initial cycles. However, the capacity of the full cell with bare Li experiences a rapid drop from the 30^th^ cycle, which can be attributed to the gradual consumption of electrolyte by the Li dendrites. The full cell with CS/DF-PEG-DF@Li can still delivers a high capacity of 82.9 mAh g^−1^ after 100 cycles, with a capacity retention rate of 60.1%. Furthermore, the Li||LFP cells with CS/DF-PEG-DF@Li anode show enhanced rate performances at high rates of 1 C and 2 C (Fig. 5e). These results confirm that the cross-linked polymer protective layer can not only effectively stabilize the Li metal anode, but also boost the charge transfer and ionic transport kinetics.

## Discussion

In summary, the CS/DF-PEG-DF gel prepared by cross-linking reaction between CS and DF-PEG-DF has been proposed to fabricate a protective layer for dendrite-free Li metal anode. The CS/DF-PEG-DF protective layer provides adequate ductility and mechanical strength *via* combining the flexibility of PEG molecules and the rigidity of chitosan molecules, which can adapt to the volume changes and stress changes caused by Li dendrite growth during the intensive cycling processes. Besides, the abundant polar groups in the CS/DF-PEG-DF protective layer can not only ensure a strong adhesion to Li metal, but also homogenize the distribution of Li^+^ at the Li/electrolyte interface. Furthermore, the ultrahigh ionic conductivity with high Li-ions transference number of CS/DF-PEG-DF SEI layer can alleviate the concentration gradients and enable rapid and homogeneous ion diffusion in the SEI layer. As a result, the CS/DF-PEG-DF@Li anode demonstrates an unprecedented cycle stability over 3200 h under a high areal capacity of 10 mAh cm^−2^ at 10 mA cm^−2^. A superior cycling stability is also achieved with an ultra-high Li utilization rate of 88.0%. When paired with high-loading cathodes, CS/DF-PEG-DF@Li anode also shows enhanced cycle stability and rate performance. We believe the principle of simultaneous superior mechanical properties and high ionic conductivity will be key factors of stable SEIs, which can shed light on interfacial design to stabilize Li metal anodes.

## Methods

### Materials

All the materials were purchased from commercial sources and used as received. dibenzaldehyde-terminated telechelic poly(ethylene glycol) (DF-PEG-DF, Mw = 2000, >95 %), Chitosan (CS, Macklin, Deacetylation 90%, Mw = 200000), Salicylic acid (SA, Aldrich, AR), Dimethyl sulfoxide (DMSO, Aldrich, Anhydrous Grade).

### Fabrication of the CS/DF-PEG-DF@Li anode

The fabrication of the CS/DF-PEG-DF@Li anode was conducted in an argon-filled glove box, where the contents of both oxygen and H_2_O are less than 0.1 ppm. The CS/DMSO solution (concentration of 1 wt%) was prepared by dissolving 0.1 g Chitosan and 0.05 g Salicylic acid in 10 g DMSO under agitated stirring for 1 h. Similarly, a certain amount of DF-PEG-DF dissolved in DMSO was configured to be a 1 wt% DF-PEG-DF/DMSO solution. Subsequently, the above two solutions were mixed with 1:1 volume ratio and strongly stirred for tens of seconds to obtain CS/DF-PEG-DF/DMSO solution. Finally, different volumes of CS/DF-PEG-DF/DMSO solution were dropped onto the surface of lithium metal within 2 min, and the DMSO was evaporated after 12 h at 30 °C, the CS/DF-PEG-DF@Li anode was obtained.

For comparison, CS@Li, DF-PEG-DF@Li anodes were also fabricated under the same conditions except for using CS/DMSO, DF-PEG-DF/DMSO solution, respectively. CS, DF-PEG-DF and CS/DF-PEG-DF films have been also prepared by drop coating CS/DMSO, DF-PEG-DF/DMSO and CS/DF-PEG-DF/DMSO solutions on glass slides, followed by drying at 30 °C for 12 h and a peeling process from glass slides. The CS/DF-PEG-DF/Li film was obtained by sandwiching the CS/DF-PEG-DF film between two Li foil for 12 h. In addition, DMSO@Li was prepared by dropping DMSO (50 μL) onto the Li foil under the same preparation conditions.

### Material characterization

Rheology analyses were performed on a Haake Mars40 rheometer with parallel plate geometry (60 mm in diameter) at 25 °C. The mechanical properties of the materials were measured by MTS Exceed E44 multi-parameter testing machine. ^1^H NMR spectra were recorded on a BRUKER ADVANCE III 600MHz/400M spectrometer. The morphology and structure of all electrodes were characterized by a field-emission scanning electron microscope (FE-SEM, Hitachi SU8010). Before testing, the cycled batteries were disassembled and rinsed with anhydrous 1,3-dioxolane (DOL) to remove the residual organic electrolyte and Li salts. Atomic Force Microscopy (AFM, Bruker DIMENSION ICON with a Nanoscope V controller) was performed to examine the roughness of Li metal anodes. X-ray photoelectron spectroscopy (XPS, ESCALab220i-XL) with 300 W Al Kα radiations was carried out to confirm the composition of the sample surface. TGA measurement was performed under N_2_ or Ar atmosphere on a NETZSCH STA 449F3 Jupiter in the temperature range of 30–800 °C or 25–350 °C (heating rate: 10 °C min^−1^). Fourier-transform infrared (FT-IR) spectra were recorded on a Nicolet IS50 FT-IR spectrometer. ^1^H solid-state nuclear magnetic resonance (SSNMR) for CS/DF-PEG-DF and CS/DF-PEG-DF/Li film was conducted by JEOL JNM-ECZ600R 600 MHz NMR spectrometer.

### Electrochemical measurements

2032 or 2025 type coin cells were used to investigate the electrochemical performances of the electrode. 1 M LiTFSI was dissolved in a mixture of DOL and 1,2-dimethoxyethane (DME, volume ratio 1:1) with 2 wt% LiNO_3_ additive was selected as the electrolyte and *Φ* 19 mm Celgard 2400 was served as the separator. For the symmetric Li||Li cell testing, two identical bare Li or CS/DF-PEG-DF@Li electrodes (thickness of 50 μm, *Φ* 13 mm or thickness of 600 μm, *Φ* 16 mm) were performed at various current density-capacity conditions to survey the Li stripping/plating behavior. To prepare the CS/DF-PEG-DF @Cu electrode, 50 μL of CS/DF-PEG-DF/DMSO solution was dripped onto the surface of *Φ* 13 μm single-sided Li/Cu anode (China Energy Lithium Co., Ltd. 50 μm Li foil was calendered onto a 10 μm Cu foil) after placing for 12 h to allow the evaporation of DMSO, the CS/DF-PEG-DF@Cu electrode was obtained. For the Li||Cu cell testing, the CS/DF-PEG-DF@Cu electrode was fully stripped Li metal up to 0.5 V (vs Li^+^/Li) under 0.5 mA cm^−2^ and then disassembled in the symmetric coin cell to obtain CS/DF-PEG-DF@Cu foil. To measure the cycling Coulombic efficiency, the CS/DF-PEG-DF@Cu or Cu foil was used as the working electrode and Li metal as the counter electrode. The fixed amount of Li was deposited on CS/DF-PEG-DF@Li or bare Cu foil, followed by stripping Li until the voltage reached up to 0.5 V at the same current density. Aurbach tests^51^ of Li||Cu cell CE were operated at 1 mA cm^−2^ with the pre-plating Li of 5 mAh cm^−2^, followed by reversible plating/stripping with 1.0 mA cm^−2^, 1.0 mAh cm^−2^, and finally stripping to 1.0 V. This CE was calculated by this equation:

1 $${CE}=\frac{{Q}_{s}+{Q}_{c}\times n}{{Q}_{p}+{Q}_{c}\times n}$$

where *Q*_*s*_ is the stripping capacity at final, *Q*_*p*_ is the plating capacity at initial, *Q*_*c*_ is the constant plating/stripping capacity for each cycle, and *n* is the cycle number. For the Li||Li and Li||Cu cells, a fixed amount (50 μL) of electrolyte was used in each coin battery. For the full cell systems, the *Φ* 10 mm LiFePO_4_ cathode electrode comes from commercial purchase, and its active substance load is 20 mg cm^−2^. The cells composed of the as-prepared cathode and Li or CS/DF-PEG-DF@Li (50 μm in thickness, *Φ* 13 mm) were cycled in carbonate-based electrolyte (1.0 M LiPF_6_ in EC:DMC:EMC=1:1:1 Vol%) at 0.5 C after 5 cycles of activation at 0.1 C in a voltage range of 2.0–4.0 V. For the lithium-sulfur battery systems, the cathode electrode was fabricated by mixing the sulfur/mesoporous carbon composite^52^, polyvinylidene fluoride (PVDF), and acetylene black with a mass ratio of 90:3:7 dissolved in N-methyl-2-pyrrolidone (NMP). The gained slurry was coated onto Carbon coated Al foil and dried at 80 °C under vacuum overnight. The sulfur loading of each *Φ* 10 mm electrode is ~7 mg cm^−2^ in this work. The electrolyte consisted of 1 M LiTFSI and 2 wt% LiNO_3_ in the mixture of DOL and DME (1:1, v/v). The dosage of electrolyte is based on the proportion of adding 20 μL electrolyte per 1 mg of active substance. The cells composed of the as-prepared cathode and Li or CS/DF-PEG-DF@Li anode (600 μm in thickness, *Φ* 16 mm) were cycled at 0.2 C after 5 cycles of activation at 0.1 C in a voltage range of 1.7–2.8 V. All the above batteries were tested at 25 °C. The electrochemical stability was evaluated through assembling the battery (Li||film||SS) by linear sweep voltammetry (LSV) from an open circuit state to 5.0 V. The LSV was carried out at a scan rate of 0.1 mV s^−1^. Electrochemical impedance spectroscopy (EIS) tests were measured on an electrochemical workstation (CHI660a, Shanghai Chenhua) in a frequency range between 10^−2^ and 10^5^ Hz. The ionic conductivity of CS/DF-PEG-DF was measured by a complex impedance method with a perturbation of 50 mV in the frequency range of 0.1–10^5^ Hz at 25–85 °C. Symmetrical stainless-steel (SS)||CS/DF-PEG-DF (swollen by 3 μL liquid electrolyte) ||SS cells were assembled to test the resistance value, and the corresponding ionic conductivity (σ, in S cm^−1^) was calculated using the Equation:

2 $$\sigma=\frac{L}{{RS}}$$

Where the L, R, and S represent the thickness (0.1 mm), ohmic resistance and area (16 mm in diameter) of CS/DF-PEG-DF, respectively. Using Li||CS/DF-PEG-DF||Li cells the lithium-ion transference number (${t}_{{{Li}}^{+}}$) was calculated by the method of Bruce and Vincent methode, according to the following equation:

3 $${t}_{{{Li}}^{+}}=\frac{{I}_{s}\left(\Delta V-{R}_{0}{I}_{0}\right)}{{I}_{0}\left(\Delta V-{R}_{s}{I}_{s}\right)}$$

Here, *R*_0_ is the interfacial resistance of the lithium electrode before the polarization, *R*_s_ is the interfacial resistance of the lithium electrode after the polarization, *I*_0_ is the current at the start of the polarization, and *I*_s_ is the steady-state current through the electrode during the polarization. Δ*V* is the polarization potential (10 mV). In-situ optical observation is performed by assembling the bare Li and CS/DF-PEG-DF@Cu anodes (the thickness of 50 μm) into a quartz cell with a transparent quartz window (Kejing, STC-Q) in a glove box. The deposition process of Li was observed and recorded with a Wi-Fi box optical microscope camera (Belona, 100X-1000X). The current density is fixed at 10 mA cm^−2^.

## Supplementary information

Supplementary Information

Description of Additional Supplementary Files

Supplementary Movie 1

## Data Availability

The data generated in this study are provided in the Source Data file. Source data are provided with this paper.

## Source data

Source Data
