# Supplementary material for: Dynamic gel as artificial interphase layer for ultrahigh-rate and large-capacity lithium metal anode
Source: Nat Commun. 2023 Jul 7;14:4018. doi: 10.1038/s41467-023-39636-6 (PMC10328938; doi:10.1038/s41467-023-39636-6)
Supplement: Supplementary file 1 — Supplementary Information [file 41467_2023_39636_MOESM1_ESM.pdf]

Supplementary Information

Dynamic Gel as Artificial Interphase Layer for Ultrahigh-rate and Large-capacity Lithium Metal Anode

Chao Chen[1], Jiaming Zhang[1], Benrui Hu[1], Qianwen Liang[1], Xunhui Xiong[1]*

[1]School of Environment and Energy, South China University of Technology, Guangzhou 510006, China

E-mail addresses: esxxiong@scut.edu.cn (Xunhui Xiong)

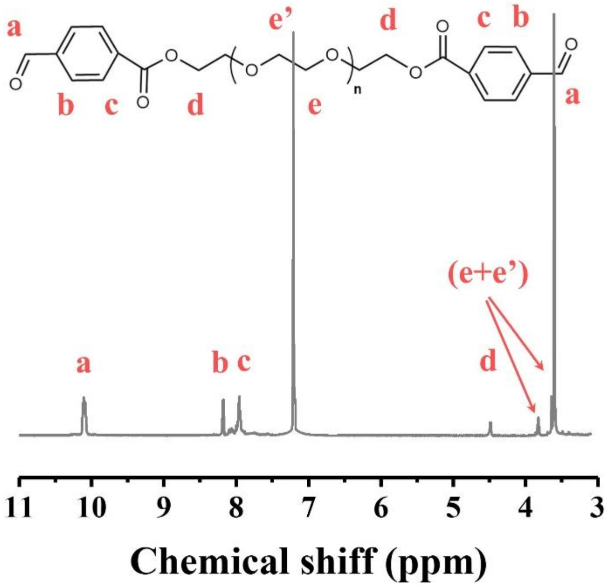

Supplementary Figure 1. ^1H NMR spectrum of DF-PEG-DF in $CDCl_3$.

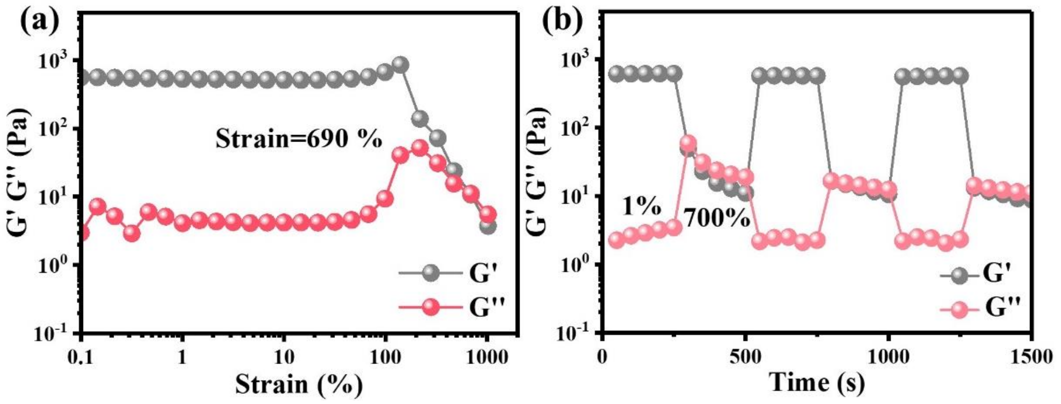

Supplementary Figure 2. (a) The strain-dependent G' and G'' of the CS/DF-PEG-DF gel by strain sweep experiments (frequency: 1.0 Hz); (b) The self-healing behavior of

the CS/DF-PEG-DF gel by the damage-healing cycles.

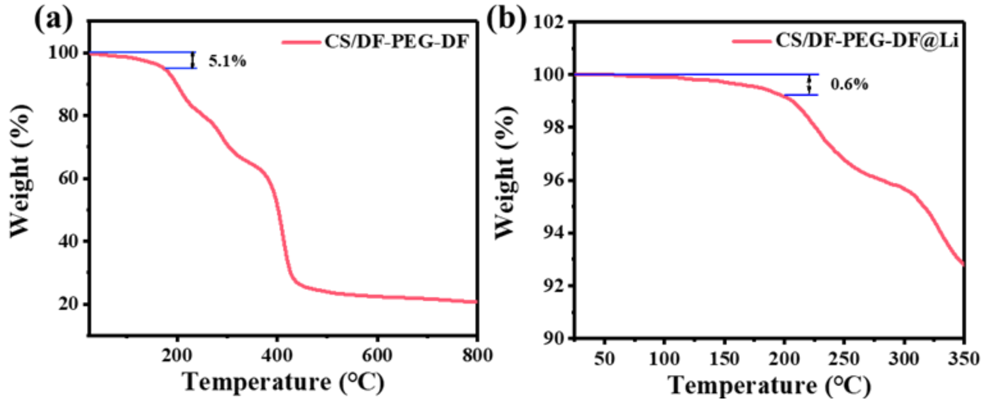

Supplementary Figure 3. Thermogravimetric Analysis (TGA) results of (a) CS/DF-PEG-DF film and (b) CS/DF-PEG-DF@Li.

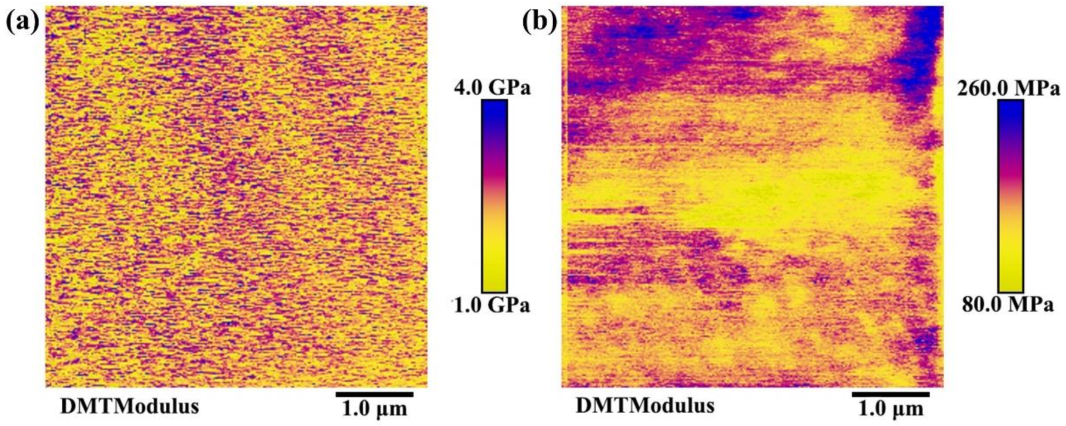

Supplementary Figure 4. Elastic modulus mapping of (a) CS and (b) DF-PEG-DF.

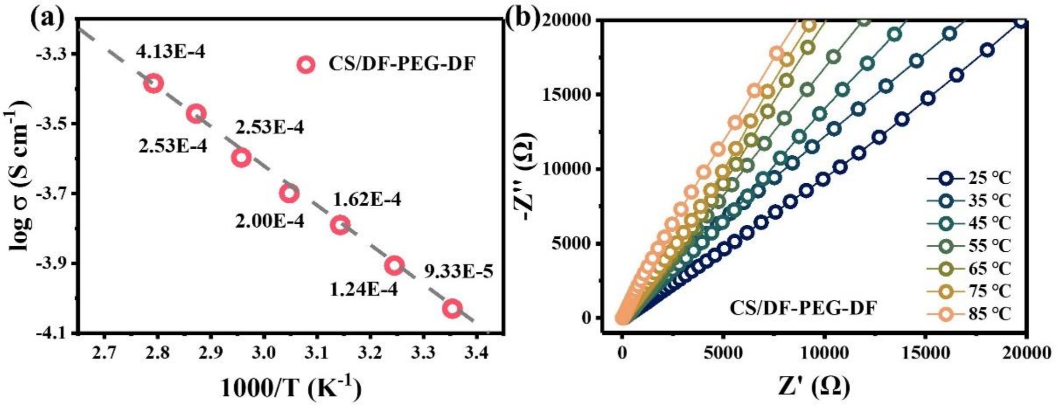

Supplementary Figure 5. (a) Temperature dependence of ionic conductivity of CS/DF-PEG-DF; (b) The impedance spectra of CS/DF-PEG-DF films at various temperatures from 25 to 85 ºC.

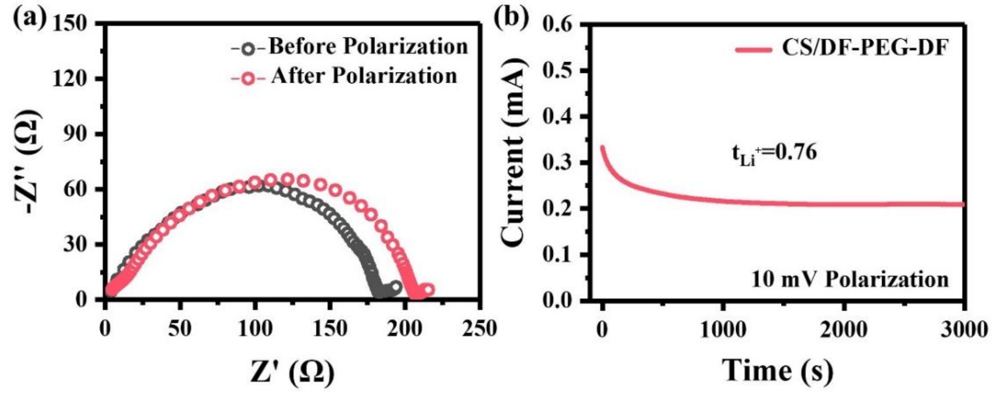

Supplementary Figure 6. (a) Electrochemical impedance spectroscopy (EIS) for the Li||Li cells before and after measured by the chronoamperometry experiment; (b)Chronoamperometry curves of Li||CS-DF-PEG-DF||Li with a static potential of 10 mV.

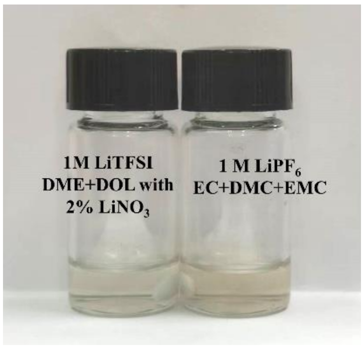

Supplementary Figure 7. Solubility of DF-PEG-DF in different electrolyte (add 0.1 g DF-PEG-DF to 1 ml electrolyte).

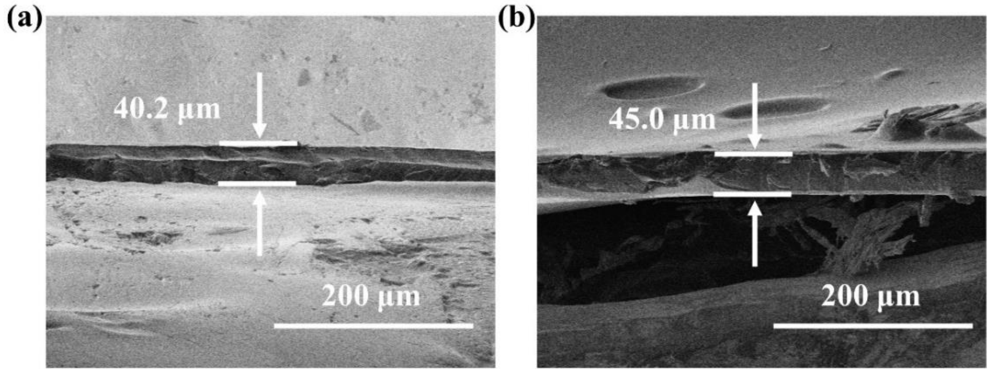

Supplementary Figure 8. Cross-section SEM images of CS/DF-PEG-DF (a) before and (b) after 5 days of immersion in ester electrolyte. It can be seen that there is no obvious swelling of CS/DF-PEG-DF film after soaking.

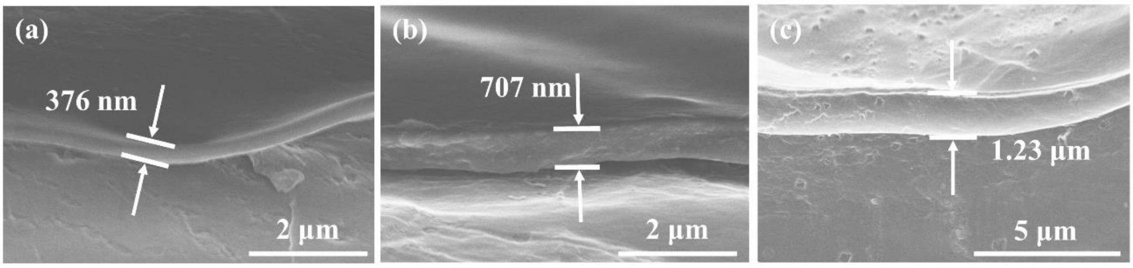

Supplementary Figure 9. SEM images of CS/DF-PEG-DF@Li fabricated by adding (a)100 μL, (b) 200 μL, (c) 400 μL of CS/DF-PEG-DF/DMSO solution on Li metal.

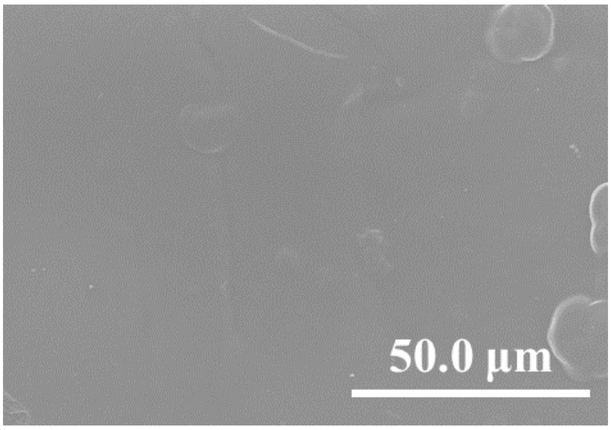

Supplementary Figure 10. SEM images of CS/DF-PEG-DF@Li anode.

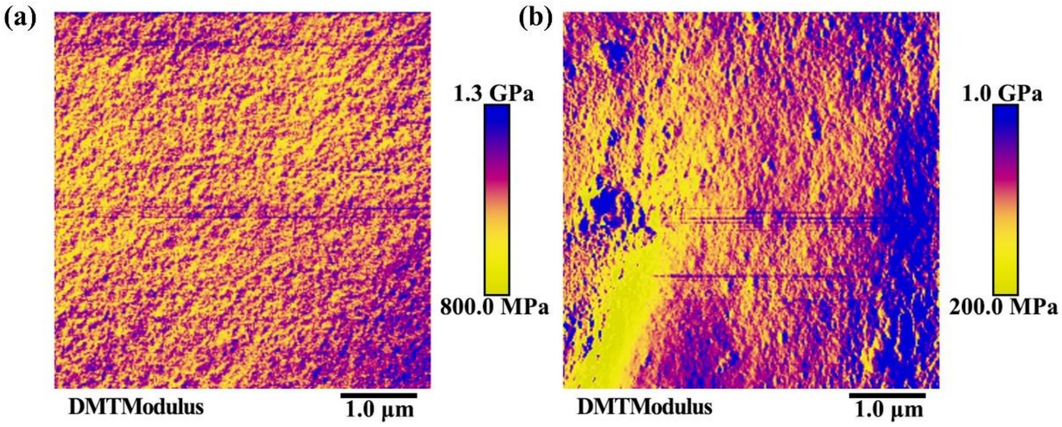

Supplementary Figure 11. Elastic modulus mapping of (a) CS/DF-PEG-DF@Li and (b) CS/DF-PEG-DF@Li after 24 h immersion in electrolyte.

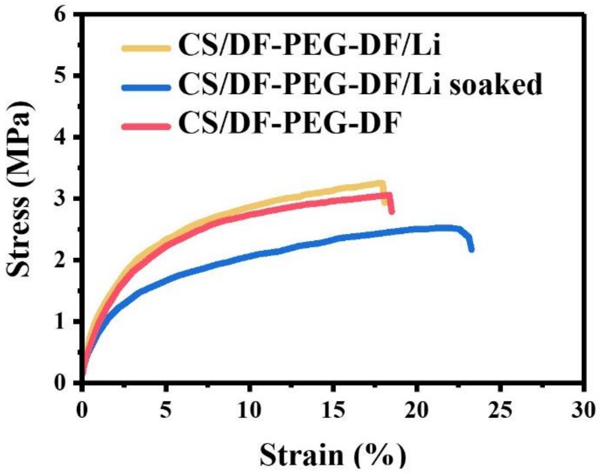

Supplementary Figure 12. Stress-strain curves of CS/DF-PEG-DF film, CS/DF-PEG-DF film after reaction with lithium metal (CS/DF-PEG-DF/Li), and CS/DF-PEG-DF/Li immersed in electrolyte for 24 h (CS/DF-PEG-DF/Li soaked).

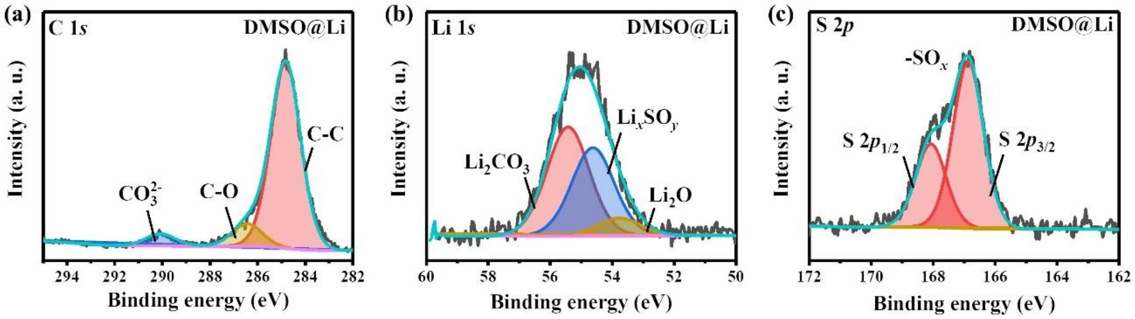

Supplementary Figure 13. XPS spectra of DMSO treated Li foil (DMSO@Li). The high-resolution XPS spectra of (a) C 1s, (b) Li 1s, and (c) S 2p for DMSO@Li.

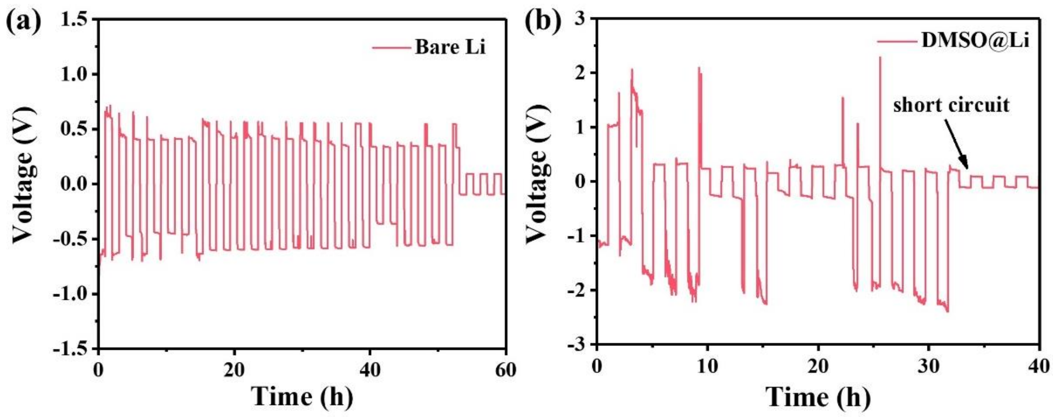

Supplementary Figure 14. Voltage profiles of (a) bare Li and (b) DMSO@Li anode

in symmetric cell at 10 mA cm^{-2} under a fixed capacity of 10 mAh cm^{-2}.

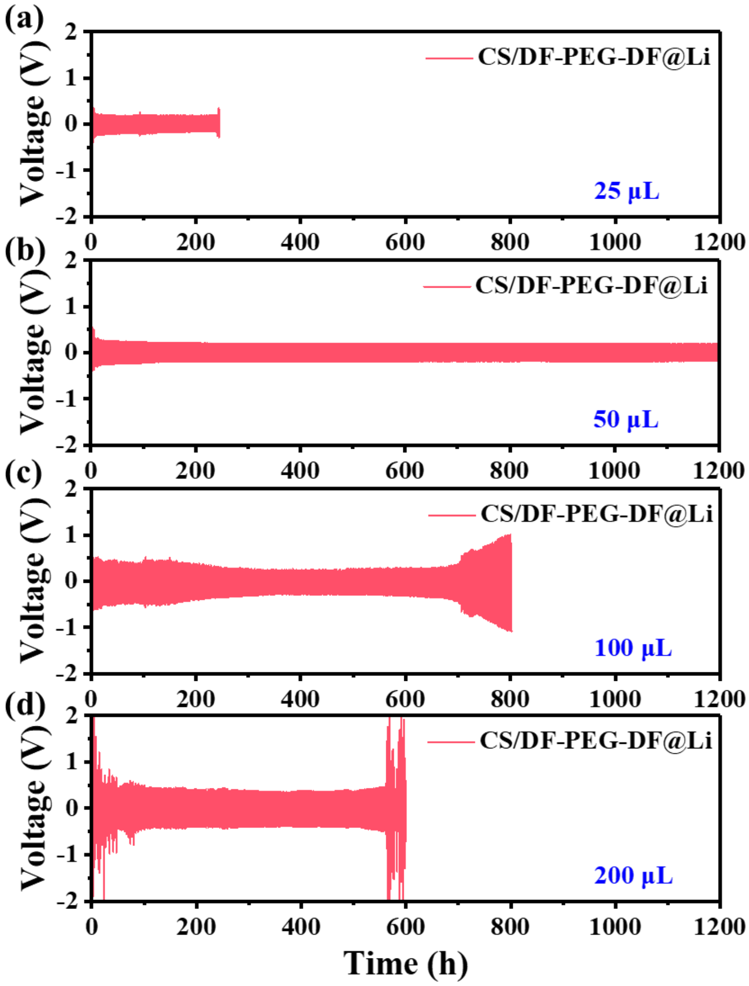

Supplementary Figure 15. (a-d) Symmetric cell cyclic stability comparison between CS/DF-PEG-DF@Li formed by adding different volumes of CS/DF-PEG-DF/DMSO solution at 10 mA cm^{-2} under a fixed capacity of 10 mAh cm^{-2}.

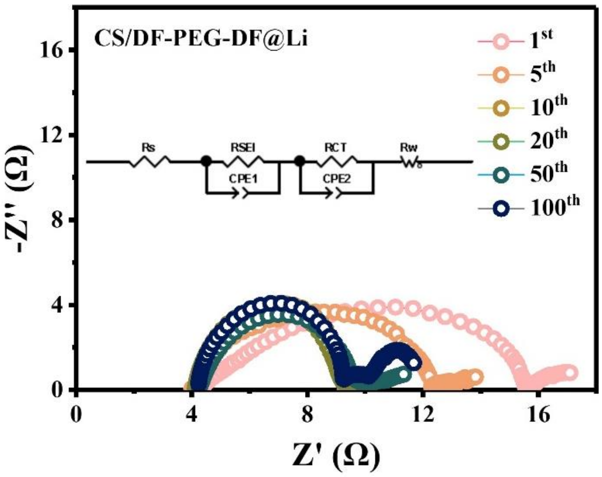

Supplementary Figure 16. EIS evolution of symmetric cells using CS/DF-PEG-

DF@Li anodes over 100 cycles.

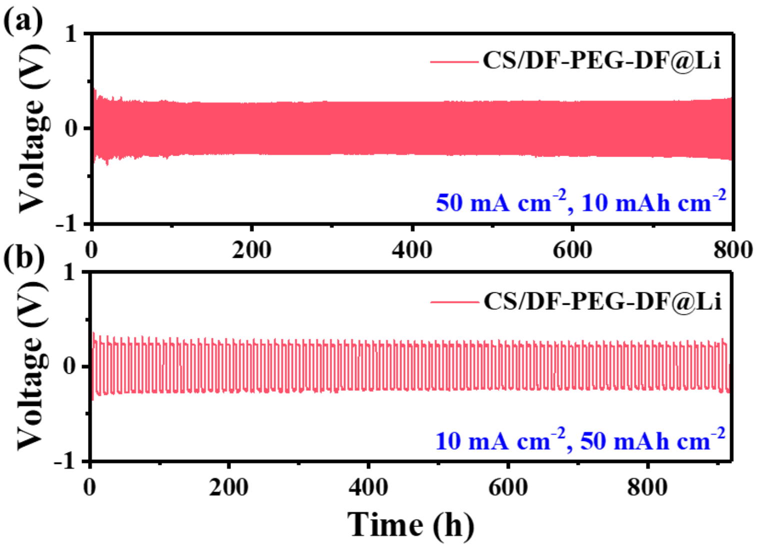

Supplementary Figure 17. Voltage profiles of CS/DF-PEG-DF@Li anodes in symmetric cell at (a) 50 mA cm^{-2} under a fixed capacity of 10 mAh cm^{-2} and (b) 10 mA cm^{-2} under a fixed capacity of 50 mAh cm^{-2}.

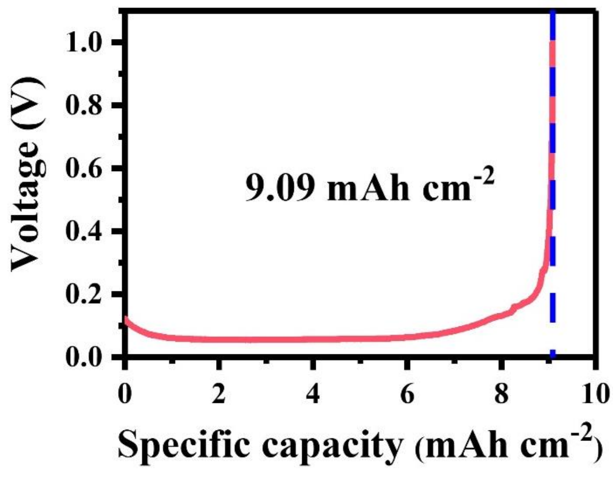

Supplementary Figure 18. Typical Li stripping curve of the thin Li anode (50 µm in thickness).

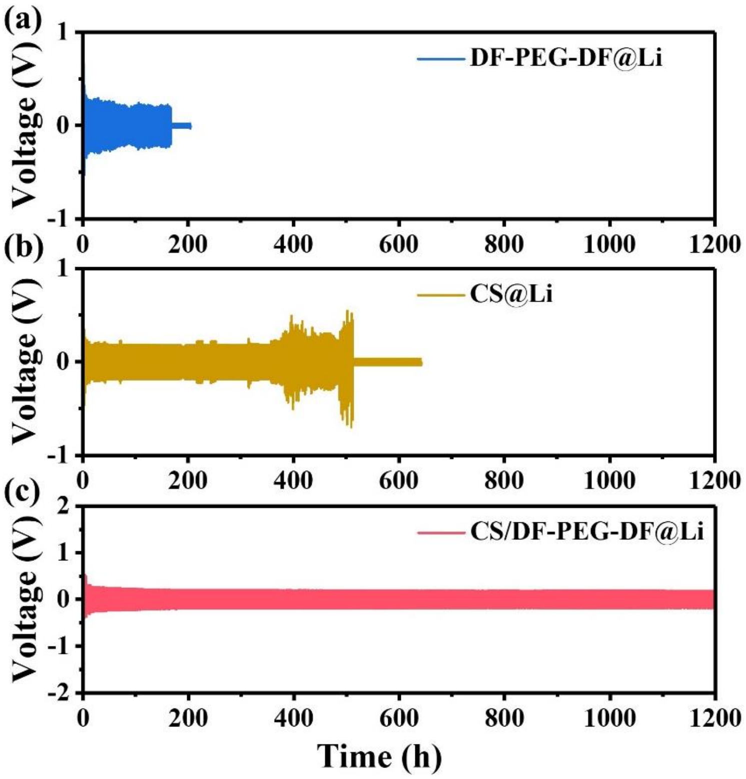

Supplementary Figure 19. Cycle stability of symmetrical cells employing (a) DF-PEG-DF@Li, (b) CS@Li, and (c) CS/DF-PEG-DF@Li at 10 mA cm^{-2} under a fixed capacity of 10 mAh cm^{-2}.

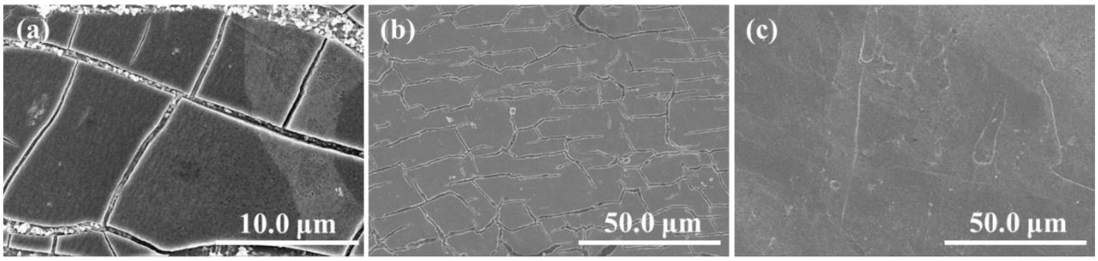

Supplementary Figure 20. SEM images of the (a) DF-PEG-DF@Li, (b) CS@Li, and (c) CS/DF-PEG-DF@Li anodes after 50 cycles at 10 mA cm^{-2}.

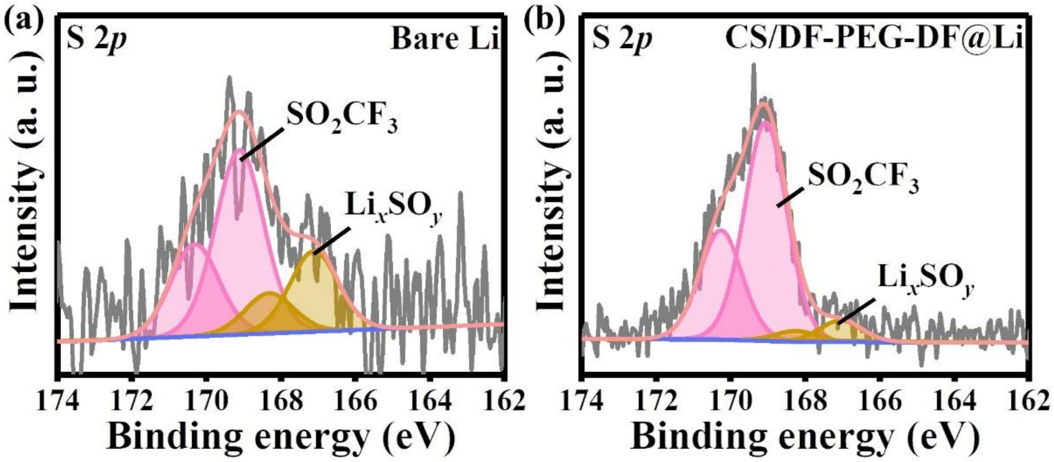

Supplementary Figure 21. S 2*p* XPS spectra of (a) bare Li and (b) CS/DF-PEG-DF@Li anodes after 10 cycles at 10 mA cm^{-2} under a capacity of 10 mAh cm^{-2}.

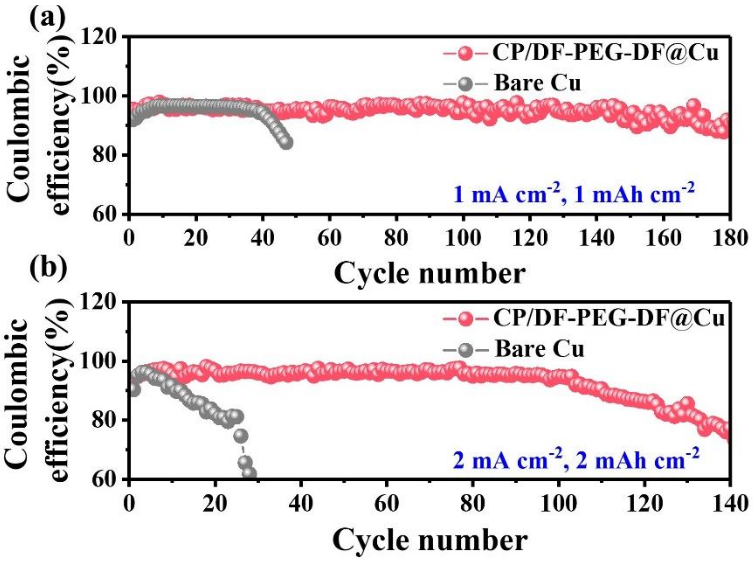

Supplementary Figure 22. CEs of bare Cu and CS/DF-PEG-DF@Cu electrodes with different capacities and different current densities in ether electrolyte.

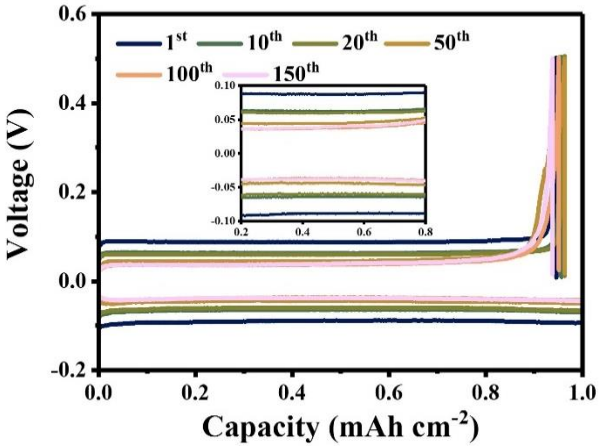

Supplementary Figure 23. Typical voltage profiles of Li||CS/DF-PEG-DF@Cu cell at 1 mA cm^{-2} under a fixed capacity of 1 mAh cm^{-2}.

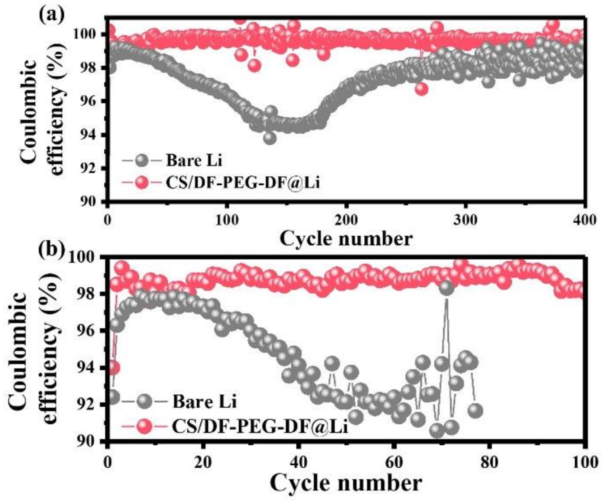

Supplementary Figure 24. CEs of (a) Li||S and (b) Li||LFP full cells.

Supplementary Table 1. Cyclic stability comparison of our CS/DF-PEG-DF@Li anode with the reported modified SEI in Li||Li symmetric cells.

Modified SEI	Current density (mA cm^{-2})	Areal capacity (mAh cm^{-2})	Cycle time (h)	Ref.
organophosphate-derived dual-layered interface	1	1	2000	1
multiwall carbon nanotubes (MWCNT)	2	2	2000	2
$Mg@C_{60}$	3	1	180	3
Ge-THF-treated Li	3	1	375	4
hybrid polyurea film	5	1	85	5
LiF	5	1	500	6
Li_2S_x	5	5	150	7
Li-Nafion/LiCl	8	1	120	8
Graphite fluoride-LiF	10	1	33	9
CNT with ZnO	10	1	100	10
Al-Li alloy/LiCl	10	5	300	11
MgO	10	5	2500	12
xPCMS-g-PEGMA/LN	10	10	2800	13
Li-Hg alloy	12	12	200	14
Li/Al_4Li_9-LiF	20	1	10	15
LiPEO-UPy layer	20	1	400	16
Li_3Sb/LiF	20	2	320	17
$Li/Li_{22}Sn_5$	30	5	66.67	18
MXene layer	35	1	420	19
**CS/DF-PEG-DF**	**10**	**10**	**3200**	**This work**
	**50**	**50**	**600**	

Supplementary Table 2. Li utilization comparison of our CS/DF-PEG-DF@Li anode with the reported literature in Li||Li symmetric cells.

Composite anode	Max areal capacity (mAh cm^{-2})	Li loading (mAh cm^{-2})	Li utilization (%)	Ref.
PI-ZnO Li	3	26.3	11.4	20
MgO@WC	3.5	20.0	17.5	21
CG host	1	4.0	25.0	22
CNF-TiN	1	3.0	33.3	23
PA-MXene-Li	20	43.0	46.5	19
CF/Ag-Li	10	21.0	47.6	24
N-doped graphene Li	12	23.6	50.8	25
D-Cu@CuSe	5	8.0	62.5	26
Li@MnZnO/CNF	10	14.3	70.0	27
Cu/Li	6	8.0	75.0	28
**CS/DF-PEG-DF@Li**	**8**	**9.1**	**88.0**	**This work**

Supplementary references

1. Liu, X., Liu, J., Qian, T., Chen, H. & Yan C. Novel Organophosphate-Derived Dual-Layered Interface Enabling Air-Stable and Dendrite-Free Lithium Metal Anode. *Adv. Mater.* **32**, 1902724 (2020).
2. Salvatierra, R. V. et al. Suppressing Li Metal Dendrites Through a Solid Li-Ion Backup Layer. *Adv. Mater.* **30**, 1803869 (2018).
3. Xu, Q. et al. Air-Stable and Dendrite-Free Lithium Metal Anodes Enabled by a Hybrid Interphase of C60 and Mg. *Adv. Energy Mater.* **10**, 1903292 (2020).
4. Liao, K. et al. Developing a “Water-Defendable” and “Dendrite-Free” Lithium-Metal Anode Using a Simple and Promising $GeCl_4$ Pretreatment Method. *Adv. Mater.* **30**, 1705711 (2018).
5. Sun, Y. et al. Tailoring the Mechanical and Electrochemical Properties of an Artificial Interphase for High-Performance Metallic Lithium Anode. *Adv. Energy Mater.* **10**, 2001139 (2020).
6. Lang, J. et al. One-pot solution coating of high quality LiF layer to stabilize Li metal anode. *Energy Storage Mater.* **16**, 85-90 (2019).
7. Jiang, Z. et al. Facile Generation of Polymer-Alloy Hybrid Layers for Dendrite-Free Lithium-Metal Anodes with Improved Moisture Stability. *Angew. Chem.-Int. Edit.* **58**,

11374-11378 (2019).

8. Li, S., Fan, L. & Lu, Y. Rational design of robust-flexible protective layer for safe lithium metal battery. *Energy Storage Mater.* **18**, 205-212 (2019).

9. Shen, X. et al. Lithium anode stable in air for low-cost fabrication of a dendrite-free lithium battery. *Nat. Commun.* **10**, 900 (2019).

10. Zhang, H. et al. Lithiophilic-lithiophobic gradient interfacial layer for a highly stable lithium metal anode. *Nat. Commun.* **9**, 3729 (2018).

11. Lu, Z. et al. Constructing a High-Strength Solid Electrolyte Layer by In Vivo Alloying with Aluminum for an Ultrahigh-Rate Lithium Metal Anode. *Adv. Funct. Mater.* **30**, 1907343 (2020).

12. Zhou, Y. et al. Redistributing Li-Ion Flux by Parallelly Aligned Holey Nanosheets for Dendrite-Free Li Metal Anodes. *Adv. Mater.* **32**, 2003920 (2020).

13. Li, S. et al. A robust all-organic protective layer towards ultrahigh-rate and large-capacity Li metal anodes. *Nat. Nanotechnol.* **17**, 613-621 (2022).

14. He, G., Li, Q., Shen, Y. & Ding, Y. Flexible Amalgam Film Enables Stable Lithium Metal Anodes with High Capacities. *Angew. Chem.-Int. Edit.* **58**, 18466-18470 (2019).

15. Wang, H., Lin, D., Liu, Y., Li, Y. & Cui, Y. Ultrahigh-current density anodes with interconnected Li metal reservoir through overlithiation of mesoporous AlF_3 framework. *Sci. Adv.* **3**, e1701301 (2017).

16. Wang, G. et al. Self-Stabilized and Strongly Adhesive Supramolecular Polymer Protective Layer Enables Ultrahigh-Rate and Large-Capacity Lithium-Metal Anode. *Angew. Chem.-Int. Edit.* **59**, 2055-2060 (2020).

17. Hu, A. et al. An artificial hybrid interphase for an ultrahigh-rate and practical lithium metal anode. *Energy Environ. Sci.* **14**, 4115-4124 (2021).

18. Wan, M. et al. Mechanical rolling formation of interpenetrated lithium metal/lithium tin alloy foil for ultrahigh-rate battery anode. *Nat. Commun.* **11**, 829 (2020).

19. Zhang, D., Wang, S., Li, B., Gong, Y. & Yang, S. Horizontal Growth of Lithium on Parallelly Aligned MXene Layers towards Dendrite-Free Metallic Lithium Anodes. *Adv. Mater.* **31**, 1901820 (2019).

20. Liu, Y. et al. Lithium-coated polymeric matrix as a minimum volume-change and dendrite-free lithium metal anode. *Nat. Commun.* **7**, 10992 (2016).

21. Jin, C. et al. Metal oxide nanoparticles induced step-edge nucleation of stable Li metal anode working under an ultrahigh current density of 15 mA cm^{-2}. *Nano Energy* **45**, 203-209 (2018).

22. Hong, S.-H. et al. Electrical Conductivity Gradient Based on Heterofibrous Scaffolds for Stable Lithium-Metal Batteries. *Adv. Funct. Mater.* **30**, 1908868 (2020).

23. Lin, K. et al. Ultrafine Titanium Nitride Sheath Decorated Carbon Nanofiber Network Enabling Stable Lithium Metal Anodes. *Adv. Funct. Mater.* **29**, 1903229 (2019).

24. Zhang, R. et al. Coralloid Carbon Fiber-Based Composite Lithium Anode for Robust Lithium Metal Batteries. *Joule* **2**, 764-777 (2018).

25. Gao, Y. et al. Polymer–inorganic solid–electrolyte interphase for stable lithium metal batteries under lean electrolyte conditions. *Nat. Mater.* **18**, 384-389 (2019).

26. Shi, Z. et al. Synergizing Conformal Lithiophilic Granule and Dealloyed Porous Skeleton toward Pragmatic Li Metal Anodes. *Small Science* **2**, 2100110 (2022).

27. Le, T. et al. Deeply Cyclable and Ultrahigh-Rate Lithium Metal Anodes Enabled by Coaxial Nanochamber Heterojunction on Carbon Nanofibers. *Adv. Sci.* **8**, 2101940 (2021).
28. Huang, S. et al. Self-Propagating Enabling High Lithium Metal Utilization Ratio Composite Anodes for Lithium Metal Batteries. *Nano Lett.* **21**, 791-797 (2021).
