# Supplementary material for: Dynamic gel as artificial interphase layer for ultrahigh-rate and large-capacity lithium metal anode
Source: Nat Commun. 2023 Jul 7;14:4018. doi: 10.1038/s41467-023-39636-6 (PMC10328938; doi:10.1038/s41467-023-39636-6)
Supplement: Supplementary file 2 — Description of Additional Supplementary Files [file 41467_2023_39636_MOESM2_ESM.pdf]

Description of Additional Supplementary Files

Supplementary Movie 1

In situ optical microscopy investigation of the electrolyte and electrode interfaces for the Li||Cu cells during Li deposition process (current density of 10 mA cm^{-2}).
